# Physical comorbidities in people with severe mental disorders: promises and future challenges

**DOI:** 10.1192/j.eurpsy.2023.48

**Published:** 2023-07-19

**Authors:** N. Sartorius

**Affiliations:** Association for the Improvement of Mental Health Programmes (AMH), Geneva, Switzerland

## Abstract

**Abstract:**

The comorbidity of mental and physical disorders is gradually becoming recognized as a major problem for health care. Its public health importance is vast and growing for a variety of reasons including the extension of life expectancy, the imperfection of currently available treatments of mental and many physical disorders and the tendency of fragmentation of medicine into ever more limited specialties. The problems related to comorbidity are also rapidly increasing in low- and middle-income countries where in addition to the issues mentioned above there is also a scarcity of means to deal with them.

The presentation will remind the audience of the complexity of the problems and draw attention to action that could be taken by public health authorities and the medical profession in the areas of teaching, organization of service and research.

**Disclosure of Interest:**

None Declared

